# Quantification of recirculation as an adjuvant to transthoracic echocardiography for optimization of dual-lumen extracorporeal life support

**DOI:** 10.1007/s00134-012-2534-z

**Published:** 2012-03-29

**Authors:** Erik P. J. Körver, Yuri M. Ganushchak, Antoine P. Simons, Dirk W. Donker, Jos G. Maessen, Patrick W. Weerwind

**Affiliations:** 1 Department of Cardiothoracic Surgery, Maastricht University Medical Centre, P. Debeyelaan 25, P.O. Box 5800, 6202 AZ Maastricht, The Netherlands; 2 Department of Cardiology, Maastricht University Medical Centre, Maastricht, The Netherlands; 3 Department of Intensive Care Medicine, Maastricht University Medical Centre, Maastricht, The Netherlands

**Keywords:** Veno-venous extracorporeal life support, Recirculation, Echocardiography, Dual-lumen cannula, Ultrasound dilution

## Abstract

**Purpose:**

Proper cannula positioning in single site veno-venous extracorporeal life support (vv-ELS) is cumbersome and necessitates image guidance to obtain a safe and stable position within the heart and the caval veins. Importantly, image-guided cannula positioning alone is not sufficient, as possible recirculation cannot be quantified.

**Methods and results:**

We present an ultrasound dilution technique allowing quantification of recirculation for optimizing vv-ELS.

**Conclusion:**

We suggest quantification of recirculation in addition to image guidance to provide optimal vv-ELS.

## Introduction

Veno-venous extracorporeal life support (vv-ELS) has increasingly been employed in adult patients with severe respiratory insufficiency [1]. Historically, vv-ELS in adult patients has required dual cannulation. A novel developed bi-caval dual-lumen cannula (Avalon ELITE™, Avalon Laboratories, LLC., CA, USA) for vv-ELS uses single site venous cannulation via the right jugular vein [2]. The cannula simultaneously drains blood from the superior and the inferior caval vein. Oxygenated blood is returned into the right atrium via the infusion port pointing towards the tricuspid valve. This approach aims at reducing the risk of insertion site bleeding, thrombosis, infection and accidental dislodgement by facilitating implantation and patient mobilization [3].

Support, however, can be negatively influenced by recirculation as a result of improper cannula position [4]. Recirculation is defined as the fraction of the oxygenated blood that exits the infusion port, and which is immediately drained back into the drainage ports. During dual-lumen vv-ELS, cannula position can be verified using transoesophageal echocardiography combined with fluoroscopy [2] or saline injection [5]. Recirculation and resultant inadequate lung assist, however, may still occur. To maintain optimal support, i.e. sufficient oxygenation and decapneization, quantification of recirculation may be crucial in correctly (re)positioning of the dual-lumen cannula. Although it has been reported in experimental and neonatal ELS using single site cannulation [6–8], to the best of our knowledge, quantification of recirculation during adult vv-ELS using single site cannulation has not been reported in the literature so far.

We describe three cases to illustrate the benefits of a technique for the quantification of recirculation as an adjuvant to transthoracic echocardiography during vv-ELS.

## Case presentations

The medical ethics committee of the Maastricht University Medical Centre approved the use of patient recordings. The technique to quantify recirculation measures changes in fluid density, meaning that it detects the dilution of blood with saline from changes in the average cross-sectional velocity of an ultrasound beam that illuminates the blood flowing through the tubing of the circuit. One ultrasonic probe is placed on the arterial inlet, whereas a second probe is placed on the venous outlet line of the dual-lumen cannula. In contrast to van Heijst et al. [7] who applied ultrasound dilution using a 5-mL saline bolus injection to quantify recirculation during vv-ELS, we in our cases used a 10-mL saline bolus. The bolus is injected into the outlet port of the oxygenator and is subsequently detected by both probes as a dilution curve. Voltage changes that correspond to the mean cross-sectional ultrasound velocity across the tubing are transmitted to the computer system. Software analysis (Transonic Systems Inc., Ithaca, NY, USA) provides a percentage of recirculation by dividing the area under the curve measured by the venous probe by the corresponding area detected by the arterial probe. Detection of changes in ultrasound velocity occurs until fluid density is in equilibrium.

**Fig. 1:**
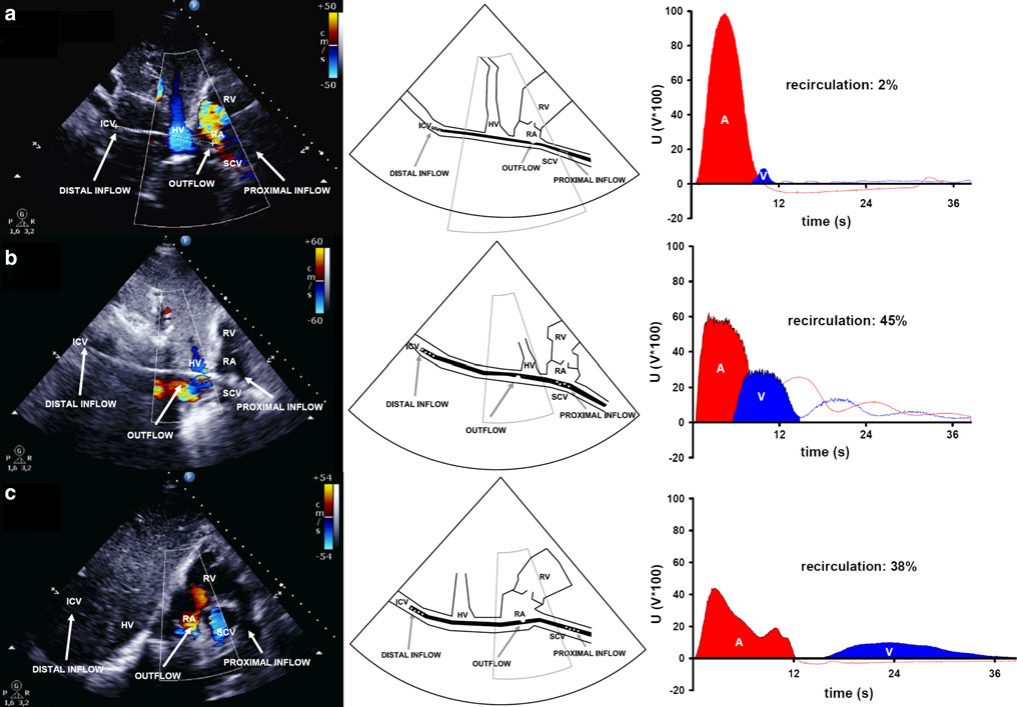
Transthoracic echocardiography (colour Doppler) shows: **a** the dual-lumen cannula (31 Fr.) situated properly in the caval veins (draining 4.3 L/min), with the outflow jet pointing at the tricuspid valve and right ventricle. The distance between the distal drainage port and the infusion port amounts to 9.4 cm (*left*). The schematic representation shows the position of the dual-lumen cannula (*middle*). Ultrasound dilution creates an arterial (A) and venous (V) dilution curve, resulting in a level of recirculation that amounts to 2 % (*right*). **b** An improperly situated dual-lumen cannula (27 Fr.). The cannula is rotated and the infusion port is located in the inferior caval vein near the hepatic vein (*left*). The schematic representation shows the position of the dual-lumen cannula (*middle*). Although ELS flow amounted to 4.7 L/min, ultrasound dilution measures a recirculation level of 45 % (*right*). **c** The bi-caval dual-lumen cannula (31 Fr.) correctly positioned. A large *blue* coloured jet within the superior caval vein is directed toward the proximal cannula drainage port and can be interpreted as drainage flow. A *red* coloured jet shows blood leaving the cannula infusion port and flowing directly toward the tricuspid valve (*left*). The schematic representation shows the position of the dual-lumen cannula (*middle*). Ultrasound dilution, however, detects a recirculation level of 38 % at a flow rate of 5.3 L/min, revealing cannula malposition (*right*). *ICV* inferior caval vein, *SCV* superior caval vein, *RA* right atrium, *RV* right ventricle, *HV* hepatic vein, *A* area under the curve measured by the arterial probe, *V* area under the curve measured by the venous probe, *U* voltages in volts × 100

In the first case, transthoracic echocardiography shows a proper cannula position, confirmed by a recirculation level of 2 % (Fig. 1a), thereby facilitating lung protective mechanical ventilation (tidal volume 3.6 mL/kg predicted body weight (PBW), positive end expiratory pressure (PEEP) 12 cmH_2_O, plateau pressure 17 cmH_2_O and 50 % oxygen fraction with an arterial saturation of 95 %).

The second case illustrates an improperly located cannula, which was confirmed by a recirculation level of 45 % (Fig. 1b). Consequently, this resulted in an undesirable increase of mechanical ventilatory support and subsequently repositioning of the cannula was needed. To maintain arterial saturation above 80 %, tidal volume was increased from 4.7 to 6.0 mL/kg PBW, with an increase of plateau pressure from 24 to 26 cmH_2_O and oxygen fraction from 45 to 100 %; PEEP 10 cmH_2_O.

The final case highlights proper cannula positioning by transthoracic echocardiography (Fig. 1c); however, mechanical ventilatory support required tidal volume to be increased from 4.7 to 6.0 mL/kg PBW to maintain arterial saturation above 80 %. Recirculation was shown to be 38 %. Thus, echocardiography alone provided a false-positive confirmation of a properly positioned dual-lumen cannula. Repositioning of the cannula allowed for a decrease of mechanical ventilatory support and return to full protective lung ventilation, while increasing arterial saturation to 94 %. Plateau pressure was diminished from 26 to 22 cmH_2_O and the oxygen fraction was halved, to 50 %; PEEP 12 cmH_2_O.

## Comments

Cannula migration can cause suboptimal vv-ELS, but resultant recirculation may remain undetected using echocardiography. Quantification of recirculation using ultrasound dilution as an adjuvant to transthoracic echocardiography proved to be a valuable tool to monitor dual-lumen cannula position during vv-ELS.

Echocardiography at the bedside has been recommended as the standard imaging technique for positioning of the dual-lumen cannula for vv-ELS. Adding fluoroscopy as an imaging modality [2], however, might affect the echocardiographic image quality as a result of the body mass index, i.e. in extreme obesity. Dolch et al. [5] described a method using transoesophageal echocardiography together with microbubbles present in saline for cannula placement visualization. Ideally, image guidance should simultaneously provide position validation and functional testing during dual-lumen cannula placement. However, as we showed in case 3, despite the confirmed correct cannula position by transthoracic echocardiography, ultrasound dilution technique revealed an inadequate lung support, having a recirculation of 38 % at a support flow rate of 5.3 L/min. In other words, when, from the clinical point of view, support proves sufficient, ultrasound imaging can fail in detecting malposition. Consequently, mechanical ventilatory support was increased and cannula repositioning needed.

Recirculation is a dynamic event that is influenced by a variety of factors, such as cannula and patient position, volume status, pump flow rate, and cardiac output [4]. Commonly, the monitoring of recirculation during vv-ELS is performed with the use of the accepted, but clinically limited and time-consuming, saturation method [6]. On the other hand, the ultrasound dilutional technique has been described as an accurate quantification method for recirculation [6, 8, 9]. Nonetheless, the latter technique has not gained widespread application during vv-ELS. We report the clinical application of quantification of recirculation in adult vv-ELS and, additionally, show the added value of this quantification in serving as an adjunct to echocardiographic imaging to improve extracorporeal lung support.

In conclusion, dilutional ultrasound provides a practical method to quantify and monitor recirculation during dual-lumen extracorporeal lung support. Therefore, the technique completes interventions that improve oxygenation and decapneization.

## References

[1] Ganslmeier P, Philipp A, Rupprecht L, Diez C, Arlt M, Mueller T, Pfister K, Hilker M, Schmid C (2011). Percutaneous cannulation for extracorporeal life support. Thorac Cardiov Surg.

[2] Javidar J, Wang D, Zwischenberger JB, Costa J, Mongero L, Sonett J, Bacchetta M (2011). Insertion of bicaval dual lumen extracorporeal membrane oxygenation catheter with image guidance. ASAIO J.

[3] Bermudez CA, Rocha RV, Sappington PL, Toyoda Y, Murray HN, Boujoukos AJ (2010). Initial experience with single cannulation for venovenous extracorporeal oxygenation in adults. Ann Thorac Surg.

[4] Pettignano R, Clark RH, Cornisch JD (2000) Principles and practice of venovenous ECMO. In: Zwischenberger JB, Steinhorn RH, Bartlett RH (eds) ECMO extracorporeal cardiopulmonary support in critical care, 2nd edn. Extracorporeal Life Support. Organization, Ann Arbor

[5] Dolch ME, Frey L, Buerkle MA, Weig T, Wassilowsky D, Irlbeck M (2011). Transesophageal echocardiography-guided technique for extracorporeal membrane oxygenation dual-lumen catheter placement. ASAIO J.

[6] Walker J, Primmer J, Searles BE, Darling EM (2007). The potential of accurate SvO2 monitoring during venovenous extracorporeal membrane oxygenation: an in vitro model using ultrasound dilution. Perfusion.

[7] van Heijst AF, van der Staak FH, de Haan AF, Liem KD, Festen C, Geven WB, van de Bor M (2001). Recirculation in double lumen catheter veno-venous extracorporeal membrane oxygenation measured by an ultrasound dilution technique. ASAIO J.

[8] Clements D, Primmer J, Ryman P, Marr B, Searles B, Darling E (2008). Measurements of recirculation during neonatal veno-venous extracorporeal membrane oxygenation: clinical application of the ultrasound dilution technique. J Extra Corpor Technol.

[9] Krivitski NM, MacGibbon D, Gleed RD, Dobson A (1998). Accuracy of dilution techniques for access flow measurement during hemo-dialysis. Am J Kidney Dis.

